# From Molecular Therapies to Lysosomal Transplantation and Targeted Drug Strategies: Present Applications, Limitations, and Future Prospects of Lysosomal Medications

**DOI:** 10.3390/biom15030327

**Published:** 2025-02-24

**Authors:** Adel A. Alhowyan, Gamaleldin I. Harisa

**Affiliations:** 1 Department of Pharmaceutics, College of Pharmacy, King Saud University, P.O. Box 2457, Riyadh 11451, Saudi Arabia; adel-ali@ksu.edu.sa; 2 Kayyali Chair for Pharmaceutical Industry, College of Pharmacy, King Saud University, Riyadh 11451, Saudi Arabia; 3 Department of Biochemistry and Molecular Biology, College of Pharmacy, Al-Azhar University, Nasr City, Cairo 11651, Egypt

**Keywords:** lysosomal diseases, proteopathy, enzyme replacement therapy, gene therapy, engineered lysosome, LDT

## Abstract

Lysosomes are essential intracellular organelles involved in plentiful cellular processes such as cell signaling, metabolism, growth, apoptosis, autophagy, protein processing, and maintaining cellular homeostasis. Their dysfunction is linked to various diseases, including lysosomal storage disorders, inflammation, cancer, cardiovascular diseases, neurodegenerative conditions, and aging. This review focuses on current and emerging therapies for lysosomal diseases (LDs), including small medicines, enzyme replacement therapy (ERT), gene therapy, transplantation, and lysosomal drug targeting (LDT). This study was conducted through databases like PubMed, Google Scholar, Science Direct, and other research engines. To treat LDs, medicines target the lysosomal membrane, acidification processes, cathepsins, calcium signaling, mTOR, and autophagy. Moreover, small-molecule therapies using chaperones, macro-therapies like ERT, gene therapy, and gene editing technologies are used as therapy for LDs. Additionally, endosymbiotic therapy, artificial lysosomes, and lysosomal transplantation are promising options for LD management. LDT enhances the therapeutic outcomes in LDs. Extracellular vesicles and mannose-6-phosphate-tagged nanocarriers display promising approaches for improving LDT. This study concluded that lysosomes play a crucial role in the pathophysiology of numerous diseases. Thus, restoring lysosomal function is essential for treating a wide range of conditions. Despite endosymbiotic therapy, artificial lysosomes, lysosomal transplantation, and LDT offering significant potential for LD control, there are ample challenges regarding safety and ethical implications.

## 1. Introduction

Intracellular trafficking pathways play a major role in health, diseases, and pathophysiology [1,2]. The loss of intracellular compartmentation and function causes serious pathological conditions such as lysosomal diseases, aging, cancer, neurodegeneration, and metabolic syndrome, as well as many other diseases [3,4]. Specifically, lysosomes are vital intracellular organelles connected with several cellular pathways, including vesicle transport, autophagy, and cell death. Thus, the lysosomal aberrations were associated with the pathophysiology of several human diseases [5,6]. In this context, lysosomal dysfunction is associated with neurodegeneration, immune modulation, heart diseases, aging, cancer, and other diseases [5,6]. Additionally, lysosomes are involved in multi-drug resistance due to the overexpression of efflux transporters, change in membrane trafficking, diminished drug uptake, and lack of drug influence [7]. Accordingly, the restoration of subcellular organelle malfunction is an attractive plan for treating genetic abnormality, malignancy, neurodegeneration, cardiovascular problems, metabolic diseases, and other diseases [4]. In this regard, small drugs and macromolecule therapeutics such as nucleic acids, proteins, peptides, photodynamic, photothermal, sonodynamic, and chemotherapeutics are suggested to reestablish the organelle’s function, including lysosomes [4]. Nevertheless, the trafficking of macromolecule therapeutics into the intracellular milieu is essential to their therapeutic action, yet they are liable to entrapment and degradation in the lysosomes [8]. Thus, the assembly of nano-drug delivery cargoes (NDCs) with lysosomal escaping capability is a hopeful tactic for efficient organelle drug delivery [9]. The surface chemistry, size, and shape augment the deposition of NDCs into particular organelles. Thus, NDCs with a large particle size are located in the cytoplasm; however, those with smaller sizes are localized into subcellular organelles [3,9]. Biological, artificial, and hybrid NDCs are hired to deliver the medicine cargo into subcellular compartments [10]. Thus, NDCs mediate receptor binding and subcellular internalization, with or without endosomal escaping capability [7,8]. The protein-sorting machinery dictates the drug delivery scientists’ impression to design NDCs for the specific targeting of lysosomes, mitochondrion, nucleus, peroxisome, endoplasmic reticulum, Golgi complex, and proteasome [3,11].

Lysosomes are an essential subcellular compartment; they contain enzymes and proteins that are important to continuing their structure and functionality [5,12]. Lysosomal enzymes perform degradative power of lysosomes; these enzymes include more than 60 acid hydrolases [5,12]. The vacuolar H+-adenosine triphosphatase (v-ATPase) is an ATP proton pump that maintains the acidic (pH ~4.5) milieu in which lysosomal enzymes function [5,12]. Lysosomal membrane proteins, including lysosome-associated membrane protein 1(LAMP1) and lysosome integral membrane protein 2 (LIMP2), act as proton pumps and promote intercellular crosstalk [5,12]. Other lysosomal proteins include v-ATPase and acid sphingomyelinase [5,12]. In fact, LAMP1 and LAMP2 make up roughly half of all lysosomal proteins and are the most prevalent lysosomal membrane proteins. Both LAMP1 and LAMP2 are essential for lysosomal biogenesis, cellular metabolism, signal transduction, and cellular homeostasis [5,12]. Lysosomal proteins are translated into the endoplasmic reticulum and transported to the trans-Golgi network transmitted directly to the endo-lysosomal system [5,12].

**Figure 1:**
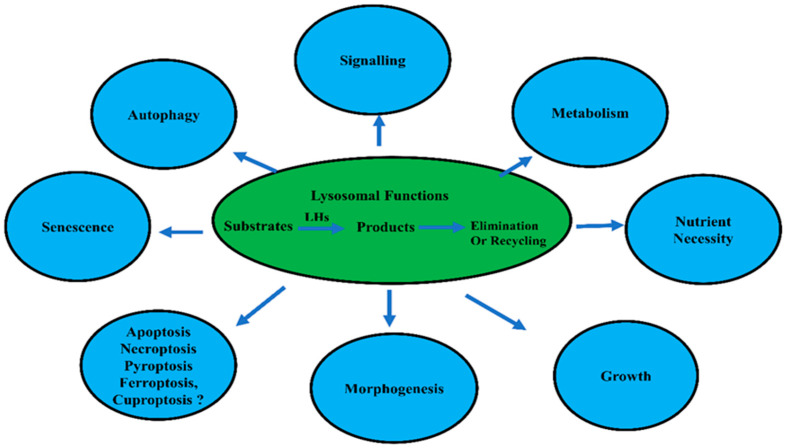
The roles of lysosomes in cell signaling, metabolism, nutrient requirements, growth morphogenesis, programmed cell death, senescence, and autophagy.

Lysosomes are called suicidal bags, cell stomachs, or cell dustbin, and they are essential to recycling both endogenous and exogenous garbage [13]. In this context, lysosomes can degrade and recycle the injured cellular and intracellular components and foreign debris; however, their accumulation induces diseases [13]. This is attributed to the acidic milieu of lysosomes, in addition to the abundance of hydrolytic enzymes such as lipases, proteases, nucleases, and other hydrolytic enzymes [13]. Accordingly, lysosomes can achieve autophagy and heterophagy to recycle the cellular debris and xenobiotics materials [13]. Lysosomes have function in cellular signal transduction, metabolic adaptation, cell proliferation, cell differentiation, and cell secretion [5,12] (see Figure 1).

**Figure 2:**
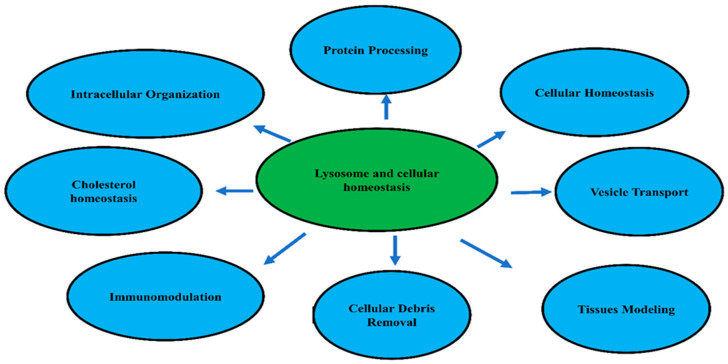
The roles of lysosomes in cellular homeostasis.

In addition, lysosomes play a role in intracellular organization and protein processing. Consequently, lysosomes are considered the directors of cellular intracellular homeostasis and tissue modeling [5,12] (see, Figure 2).

Together, lysosomes serve as the site for the recruitment and activation of the mammalian target of rapamycin (mTOR), which controls cell metabolism, nutrient requirements, growth, differentiation, programmed cell death, senescence, and aging. In addition lysosomes serving as the destination of several trafficking pathways, including autophagy, endocytosis, and phagocytosis [5,6]. In this regard, ionized calcium is released from lysosomal calcium channels to regulate vesicle trafficking, cell signaling, and the fusion of lysosomes with phagosomes and endosomes [5,6]. Moreover, the leakage of lysosomal cathepsins, calcium, iron, and redox signals contributes to several pathophysiological changes [5,6]. Thus, lysosome malfunction is implicated in inflammation, oncogenesis, autoimmune diseases, cardiovascular diseases (CVD), neurodegenerative diseases (NDDs), aging, and several diseases [5,6]. As a result, lysosomal acidification, lysosomal cathepsins, mTOR, lysosomal biogenesis, and autophagy are potential targets for drug discovery [5,6]. Specifically, malignant transformation, cancer pathogenesis, progression, and antigen presentation are linked to lysosomal dysfunction [4]. In this regard, cancer cell growth is associated with the modulation of lysosomal activity to meet the needs of tumor cell proliferation, angiogenesis, and metastasis [5,14]. Thus, the abnormal liberation of lysosomal enzymes is a motive force for the metastasis of cancer cells. Moreover, lysosomes are a crucial factor for cancer cells’ multidrug resistance to anticancer drugs [5,15]. Also, the abnormal modification of the histocompatibility complex by lysosomes in malignant cells leads to abnormalities in autophagy and induces tumor immune escape [5,16]. Similarly, in autoimmune diseases, there is a defect in lysosomal biogenesis, acidification, and cathepsin activity [5,12]. This is accompanied by the enlargement of lysosomes with the instability of lysosomal membranes and cellular aberration [5,14].

Importantly, the lysosomes are linked with autophagy which is associated with numerous human diseases pathophysiology [17,18]. However, ample autophagy processes are involved in cellular metabolism, morphogenesis, apoptosis, oncogenesis, inflammation, and other pathological conditions [17]. Particularly, the relationship between autophagy and tumorigenesis depends on specific cancer types, stages, metabolic changes, tumor-promoting immune responses, and environmental conditions [17,19,20]. Furthermore, autophagy mediates tumor-killing; however, autophagy-related proteins and transcriptional factors prevent tumor progression [17,21]. On the contrary, autophagy might mediate cancer’s pro-survival mechanism to facilitate malignant cells to adapt to different stresses [17,19]. The abundance of lysosomes in senescent cells might push autophagy; thus, the triggering of autophagy by therapeutic interventions could induce senescent cell death [22]. Lysosomal impairment is connected with subcellular homeostasis interruption, as well as the liberation of lysosomal enzymes that trigger cell death [23]. Accordingly, the rescue of lysosomal function and biogenesis is vital to maintain cellular functions [23]. For instance, NDDs such as Alzheimer’s disease and Huntington’s disease are characterized by the accumulation of abnormal proteins due to lysosomal dysfunction and a lack of autophagy machinery [17,24]. In this context, early-stage autosomal recessive Parkinson’s disease is characterized by a mutation in lysosomal ATPase. This accelerates the accumulation of α-synuclein in brain tissues [17,25]. Likewise, lysosomal aberration is associated with the deleterious effect related to several health problems [17,25].

Together, lysosomal modulation is implicated in health and diseases; thus, understanding lysosomal functions could provide a new approach to the discovery of novel therapeutics for the prevention and eradication of diseases [5,6]. In this regard, the restoration of lysosomal abnormalities could resolve the dilemma of health problems related to lysosomal malfunction [5,26]. In this context, the targeting of lysosome acidification, calcium, enzymes, mTOR, biogenesis, and autophagy is promising for the treatment of lysosome-connected pathology [5]. Additionally, the restoration of lysosomal defects by small-molecule therapy, macro-therapy, and lysosomal transplantation is a promising approach for controlling the disease’s dilemma [27,28]. Moreover, senotherapies can rescue lysosomal function [22]. Also, endosymbiosis could be harnessed in lysosomal transplantation and the production of artificial lysosomes for medical applications in the treatment of lysosomal-related diseases [29].

Lysosomal storage diseases (LSDs) and acquired lysosomal diseases (LDs) requires lysosomal drug targeting (LTD) [4]. In this context, numerous ligands such as mannose-6-phosphate (M-6-P), transferrin (TF), growth factor (GF), and low-density lipoproteins imported by clathrin-mediated endocytosis (CME) to achieve the lysosomal drug targeting [3,30]. Extracellular vehicles (EVs) were reported as tools for lysosomal drug delivery and lysosomal transplantation for the management of lysosomal defects [31].

This review aims to highlight comprehensive, updated, and novel information about the lysosomal dual functions in health and disease. Moreover, the lysosomal therapeutic from small molecules to macro-therapeutics and lysosomal transplantation were discussed in this research article. Different search tools such as PubMed, Google Scholar, and other scholarly databases were leverage in the data collection in this review.

## 2. Lysosome-Associated Diseases

**Figure 3:**
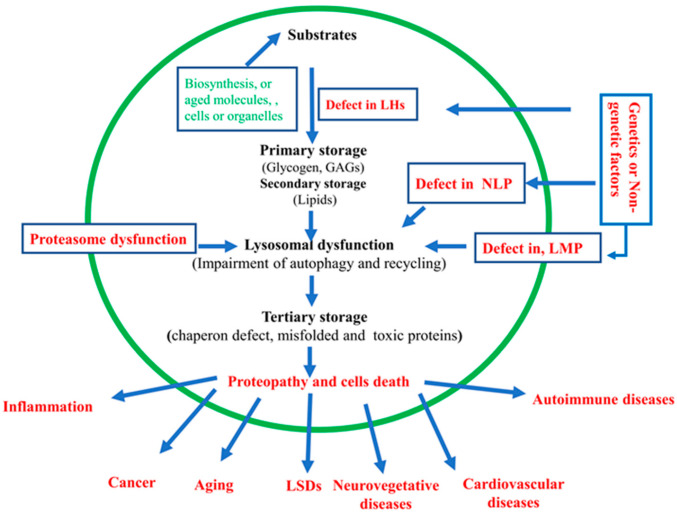
Lysosome-related diseases.

Lysosomal malfunction is implicated in inflammation, oncogenesis, autoimmune diseases, cardiovascular diseases, NDD, aging, and other diseases [5,6] (see Figure 3).

### 2.1. Lysosomal Diseases (LDs)

LSDs comprise more than 70 inborn errors of the metabolism and these disorders occur in 1 in 5000 live births [27]. The reason for LSDs is defects in genes related to lysosomal enzymes and lysosomal homeostasis. These defects cause abnormal storage and the degradation of essential complex cellular macromolecules such as lipids, proteins, carbohydrates, or their combination [27]. This can trigger oxidative stress, autophagy dysfunction, inflammation, altered biomembranes transport, and multiple pathophysiological alterations [32]. Commonly, the dysfunction of autophagy machinery in the induced accumulation of enzyme substrates within lysosomes [32]. This causes a variety of pathological changes, such as oligosaccharide storage, sphingolipidosis, mucopolysaccharidosis (MPS), and mucolipidosis [32]. Thus, LSDs are either inherited metabolic disorders or acquired LDs associated with the accumulation of amyloids and other cellular debris in nerve cells. Accordingly, about 70% of LD patients display neurodegeneration manifestation [27]. Furthermore, many lysosomal acid lipase deficiency patients have significant coronary artery disease, valvular disease, and cardiac hypertrophic and dilated cardiomyopathy [32]. Several studies documented the connection between many LDs, aging, Parkinson’s, Alzheimer’s, cancer, and other diseases. This is attributed to an imbalance in the lysosome–autophagy axis [33].

Treatment strategies for LDs include small-molecule drugs such as pharmacological chaperones (PCs) and substrate reduction therapy (SRT), as well as macromolecule therapeutics such as enzyme replacement therapy (ERT), gene therapy, and gene editing technology. Besides extracellular vesicles, organelles, cells, and organ transplantation [27,28], there is clinically approved therapy for several lysosomal diseases, which is enzyme replacement therapy [27,28,34]. These enzymes were produced utilizing recombinant DNA technology to be periodically injected life-long into LD patients [27,34]. This is the first clinically approved ERT for Gaucher disease (β-glucocerebrosidase deficiency). Afterward, other enzyme replacement therapies were clinically approved for LDs.

### 2.2. Lysosomes in CVD Pathophysiology

The lysosomal dysfunction in CVDs was reported by ample studies [5]. Indeed, in myocardial ischemia, increased autophagy prevents cardiomyocytes from dying in hypoxia [5]. On the other hand, autophagosome elevation was reported to lead to cardiomyocyte death [5]. Similarly, an impaired autophagic flux and low autophagosome clearance promote heart failure, atherosclerosis, and maladaptive post-infarction remodeling [5]. It has been reported that cathepsin D, as one of the lysosomal proteins is reduced in human heart diseases. However, cathepsin D is one of the major protease enzymes in the lysosome. Thus, lysosomal dysfunction elevates the autophagic vacuoles that cause heart cell death [35]. In the myocardial infarction situation, the induction of cathepsin D was demonstrated to mediate cardiac tissue remodeling and protect against heart failure [5]. Moreover, mutations in the LAMP2 gene cause an increase in the amount of vacuoles in the heart cell due to defects in autophagosome maturation [5]. Also, anticancer medication causes cardiotoxicity due to a lack of cardiac autophagy [36]. This results in the accumulation of oxidized debris accounts for cardiomyocyte injury induced due to lysosomal defects [36]. Additionally, the defective incorporation of autophagosomes with lysosomes decreases the removal of autophagosomes with an elevation of autophagic vacuoles in cardiac muscle cells [37]. Likewise, reduced lysosomal activity to cardiac muscle ischemia–reperfusion injury was documented [37]. In this context, certain hypoxia-inducible pro-death protein is up-regulated due to the diminution of lysosomal function, and accumulation of autophagosomes leading to the death of cardiomyocytes [37].

Additionally, proteasomal dysfunction is linked with human heart disease; thus, the increased concentrations of ubiquitinated proteins were observed in CVDs [37]. Aggregated ubiquitinated coupled proteins can be degraded in lysosomes; however, individual polyubiquitinated proteins would be in the proteasome [37]. Thus, inadequate trafficking to the proteasome or proteasome dysfunction reduce the removal of aggregated ubiquitinated proteins by lysosomes [37]. Consequently, the rebuilding of lysosomal function can reduce proteotoxicity as a therapeutic approach for CVDs [37]. Thus, the supplementation with lysosomal enzymes could treat CVDs [36]. Likewise, the development of the genetic program is a promising plan for the renewal of the lysosomal functions and treatment of CVDs [37].

### 2.3. Lysosomes in Tumors Pathophysiology

Frequently, cancer cells activate metabolic pathways to facilitate malignant cell proliferation and metastasis. These changes are associated with dramatic alterations in lysosome activity [5,14]. In this context, tumor cells increase nutrient hunting to survive in unfavorable environments [5,38]. Furthermore, the overexpression of certain lysosomal enzymes was correlated with carcinogenesis [5,15]. Likewise, the abnormal activation of some oncogenes was found to be triggered by the overexpression of lysosomal enzymes [5,15]. For example, lysosomal proteins implicated in oncogenesis, cell proliferation, and cancer metastasis were overexpressed in adenocarcinoma, melanoma, breast cancer, and other tumor types [5,14].

Additionally, the increase in lysosomal enzyme expression is associated with the development of anticancer drug resistance [5,14]. Increasing nutrient-scavenging by cancer cells activates mTOR signaling that activates a certain kinase that promotes the cell synthesis of amino acids, glucose, nucleotides, and fatty acids as essential elements for cell proliferation [5,38]. This increases catabolic and anabolic mechanisms that facilitate the proliferation of tumors. In this regard, the mTOR complex 1 (mTORC1) is dysregulated and fails to keep the balance between different catabolic and anabolic processes associated with uncontrolled cell growth [5,14].

Furthermore, because the upregulated autophagy process breaks down substances derived from epithelial tissue, it helps with growth cell migration [5,15]. Malignant-cell-enriched lysosomes that release cathepsins, heparinase and other enzymes promote cancer cell invasion and angiogenesis [5,14]. Additionally, a key factor in the escape of cancerous cells from the host immune system is lysosomal dysfunction. However, major histocompatibility complex-I presentation and antigen presentation are mediated by lysosomal proteins [5,39]. For example, the degradation of histocompatibility complex-I by lysosome through an autophagy-dependent process is associated with the decreased expression of histocompatibility complex-I on the pancreatic cancer cell surface [5,39]. Therefore, autophagy suppression and histocompatibility complex-I co-localization with lysosomes were linked to raising T-cell response and restoring histocompatibility complex-I levels in cancerous conditions [5,39]. In addition, immune checkpoints like integrin-associated protein cluster of differentiation 47 (CD47), programmed cell death-ligand-1, and cytotoxic T-lymphocyte-associated protein are degraded and presented by lysosomes [5,15]. Programmed cell death ligand interacts with programmed cell death on T cells to evade T-cell-induced immunosurveillance [5].

Likewise, it has been observed that tumor-infiltrating T lymphocytes accumulate more depolarized mitochondria when mitophagy activity is reduced [5,40]. The persistent decrease in metabolic sufficiency as a result of defective mitophagy leads to toll-like receptor exhaustion [5,40]. When combined, these elements lead to a low immune response in cancer [5,14]. Moreover, these changes are responsible for cancer invasion due to the instability of lysosomal membranes [5,14].

Taken together, in malignant cells, there is an increase in lysosomal membrane permeability, which provides therapeutic strategies; therefore, it is possible to cancer therapeutic strategies that target lysosomes [5,14]. Indeed, lysosomes contribute to anticancer drug resistance via the sequestration of internalized drugs and preventing their action inside the cancer cells [5,41].

### 2.4. Lysosomes in NDD Pathophysiology

NDDs include Huntington’s disease, Parkinson’s disease, Alzheimer’s disease, multiple sclerosis, lateral sclerosis, Prion diseases, and other NDDs [42]. NDDs are nervous system diseases initiated from the deposition of transformed macromolecules, damaged organelles, or injured cells in the nervous system [42,43]. Several studies reported lysosomes as key contributors to the pathology of NDDs; however, lysosomes are also involved cell signaling, neuroinflammation, ferroptosis, and elimination of protein aggregates [43]. Thus, the understanding of the lysosomal role in degrading cellular waste underscores its importance in health, diseases, and drug discovery for NDD therapies [43]. Specifically, lysosomal malfunction impairs autophagy machinery, which is responsible for the eradication of damaged organelles, misfolded proteins, and external debris [44]. This results in proteopathies and the accumulation of abnormal proteins and promoted NDD pathogenesis [5,45].

It has been documented that increased oxidative stress triggers redox signals that mediate the damage of v-ATPase activity with the loss of lysosomal acidification impairment of autophagy machinery and the accumulation of toxic proteins [5,46]. Nerve cells are sensitive to oxidative stress due to high oxygen consumption, increased metabolic activity and weak free radical scavenging capacity [47]. Oxidative damage and inflammation mediate the accumulation of misfolded proteins as major risk factors for NDDs [42]. There are ample studies documenting the presence of misfolded-protein NDDs [48]. In this context, several types of misfolded protein aggregates are present in the cells of NDD patients; for example, Lewy bodies are present in Parkinson’s disease [48]. In such cases, alpha-synuclein and other misfolded proteins are the main components of Lewy bodies that are connected with Parkinson’s disease [5,45]. Alpha-synuclein diminishes lysosomal degradation power. This increases protein deposition in neuron synapse dysfunction and neuronal cell death [5,45]. Likewise, amyloid plaques are associated with Alzheimer’s disease, as well as the presence of Pick bodies connected with frontotemporal dementia. Furthermore, prions are the main protein deposit in prion diseases and transmissible spongiform encephalopathy [48]. These bodies are composed of fibrous proteins that induce neuronal damage with neuronal function loss and cell death [42]. However, the conversion of globular proteins into fibrous is associated with the function loss of proteins and the production of toxic proteins that induce proteopathies [48].

Thus, protein toxicity is the main mechanism of NDD pathology; however, fibrous proteins form insoluble aggregates that accumulate in neurons and induce proteinopathies [48]. Toxic proteins in NDDs are disease-specific agents; for example, amyloid-beta and tubulin associated unit (tau) are connected with Alzheimer’s disease. However, alpha-synuclein is related to Parkinson’s disease, and huntingtin is connected with Huntington’s disease [49]. The activation of certain enzymes such as gamma-secretase, beta-secretase and transglutaminases, besides the lack of chaperones, is associated with the production of proteotoxic proteins [50]. In this regard, brain cells overexpress transglutaminases that interact with amyloid-beta, tau, alpha-synuclein, and huntingtin to produce a malfunctioning misfolded protein that is resistant to lysosomal proteolysis [49].

Heat shock proteins preserve proper cellular protein homeostasis; however, chaperones are responsible for the restoration of protein misfolding. Likewise, protein degradation pathways destroy unnecessary and dysfunctional proteins [51]. The activity of this machinery is declining due to aging and is considered a risk factor for NDDs [51]. In this context, chaperone-mediated autophagy deficiency causes proteinopathies and the preposition of protein aggregates in nerve cells [5,52]. Moreover, the decrease in lysosome–proteasome machinery, the protein–ubiquitin system, is proposed as one cause of proteopathies and the development of toxic protein species [53,54]. These toxic proteins impair the neuronal membrane function, damage genetic materials, cause mitochondrial dysfunction, disrupt axonal transport machinery, and trigger the apoptosis of nerve cells [53,54].

Lysosomal degradation depends on cathepsin expression, and there is growing evidence about the reduction in cathepsins in NDDs [5,45]. However, cathepsin D is the hydrolase responsible for degrading certain genes involved in NDD pathogenesis [5,45]. For example, the production of Aβ peptides is attributed to cathepsin D [5,55]. Additionally, elevated cathepsin B is correlated with Alzheimer’s disease [5,56]. Thus, cathepsins could be a target for the treatment of NDDs [5,56]. Additionally, the transcription factor EB (TFEB) is essential for controlling autophagy and lysosomal biogenesis. The activity of TFEB is regulated by mTOR-mediated phosphorylation-related lysosomal signals [57]. Accordingly, TFEB can bring the intracellular clearance of pathogenic factors in NDDs. Hence, TFEB is a good therapeutic target for the discovery of drugs to control NDDs [57]. When TFEB is activated genetically, it partially restores the breakdown of misfolded proteins and slows the progression of common NDDs like Alzheimer’s, Parkinson’s, and Huntington’s [5,58].

Taken together, oxidative stress is one of the main causes of neurodegeneration at the subcellular level, including lysosomes, mitochondria, and other organelles [43]. Up to date, NDDs are progressive diseases and incurable, thus developing therapies for such diseases is still challenging. Gaps in the fundamental molecular, subcellular, and cellular mechanisms of NDDs are the cause of this [43]. Accordingly, the careful understanding of the roles of lysosomes in NDD pathology, at the level of the molecular, and organelles is vigorous to develop novel NDD medicines [43]. In this context, the thought of chaperone-mediated autophagy is a potential target for the discovery of NDD therapy [51]. Thus, therapeutic methods that modulate chaperone-mediated autophagy could restore the lysosome degradation ability to prevent NDDs [59].

Notably, therapeutic interventions that restore protein-degrading systems by different autophagy pathways could prevent nerve cell damage [51]. The success of proteasome inhibitors in cancer therapy encourages the development of NDD medicines [51]. Autophagy machinery eradicates the toxic proteins besides damaged subcellular organelles, thus they are cellular friends or enemies. Therefore, the dual role of autophagy machinery should be considered during the design of autophagy-acting medicines [51].

### 2.5. Lysosomes and Aging

Lysosomes influence cellular aging by regulating autophagy, heterophagy, and storage capacity [60]. Lysosomal signals, acidic milieu, enzymes, biogenesis, and the role of lysosomes in autophagy machinery are connected with the role of lysosomes in age pathophysiology [5]. However, lysosomes are essential for ample cellular jobs, including apoptosis, repair, development, differentiation, and reactions to food and stress [60]. Thus, lysosomes orchestrate intracellular digestion machinery that eliminates undesirable materials such as damaged macromolecules and organelles, such as common debris accumulated during cellular aging [60]. The molecular characteristics of aging include proteopathies, genomic instability, telomere attrition, and epigenetic changes [61]. Likewise, the interruption of membrane transport machinery, dysbiosis of the microbiome, deregulated nutrient sensing, and mitochondrial dysfunction, alongside, cellular senescence, stem cell collapse, and the accumulation of cellular waste are indicators of cellular aging [61,62].

Frequently, cellular aging is connected to the malfunction of lysosomal enzymes, reduced acidification, and disrupted calcium regulation [60]. Moreover, the formation of highly cross-linked aggregates of oxidized proteins that interact with carbohydrates, lipids, nucleic acids, and metals forms advanced glycated end products (AGEs), lipofuscin, and other damaged compounds [60]. Cellular aging is caused by the accumulation of lipofuscin and AGEs such as particles in the cells [63]. This resulted in the imbalance of catabolism and anabolism due to the lack of lysosomes as key players in intracellular digestion [64].

Indeed, lysosomes are the key players in autophagy that deal with endogenous materials; however, xenobiotic agents are degraded by a process called ’heterophagy’. There are three types of autophagy and heterophagy: macroautophagy, microautophagy, and chaperone-mediated autophagy [64]. Deficiencies of autophagy machinery due to lysosomal defects cause the development of cellular aging [64]. Consequently, cellular aging results from the deposition of damaged biomolecules and organelles due to redox imbalance and lysosomal malfunction [64,65]. The debris of biomolecules can induce pathophysiological changes at the molecular, organelle, cellular, tissue, and organ levels [64]. Commonly, aging is associated with cancer, diabetes, CVDs, pulmonary diseases, kidney diseases, NDDs, and other diseases [66].

At the proteomic level, the loss of proteostasis as a result of lysosomal aberration leads to a lack of proteolysis and proteopathies due to misfolding proteins [67]. In this context, the autophagy–lysosomal pathway is usually disrupted in aging-related diseases with the accumulation of protein aggregation [67]. Additionally, epigenetic modifications contribute to lysosomal proteolysis. Thus, understanding cellular aging requires an understanding of the crosstalk of epigenetic processes during chromatin remodeling, the post-translational modification of histones, and DNA methylation patterns [17,68]. In this regard, there are alterations in gene expression by modulation methylation patterns in terms of the hypomethylation or hypermethylation of DNA [17,68]. The aberrant methylation was recognized in Parkinson’s disease in terms of hypermethylation at 928 cytosine residues [17,68]. This resulted from the proteopathies of DNA methyltransferases-1 with decreasing the clearance of the aggresome [17,69]. On the contrary, DNA methyltransferase-1 knockdown strengthens autophagy and induces Huntington’s disease [17,69].

Upon aging the disruption of autophagy machinery contributes to aging-related diseases that modulate the expression of certain genes epigenetic, genetic, and proteomic alteration [17,70]. This is the possible autophagy mechanism for understanding aging-related pathology and the development of therapeutic approaches [17,70]. However, autophagy induction mediates the clearance of toxic proteins and intracellular debris associated with cellular aging progression [67]. These products interfere with abnormal cell signaling and modify cellular metabolism, growth differentiation, morphology, and mortality [63].

Membrane instability, the loss of lysosomal acidification, organelle swelling, and the release of active cathepsins that cause protein turnover, mitochondrial dysfunction, and cell death are all signs of cellular aging brought on by lysosomal dysfunction [71]. This resulted in oxidative stress, insufficient autophagic clearance, and malfunctioning subcellular organelles [71]. Taken together, elderly cell damage is confirmed by the presence of damage to biomolecules, organelle dysfunction, and lysosome impairments [71]. Therefore, enhancing lysosomal functions with medicines can delay the progress of cellular aging by rebuilding lysosomal integrity [71].

Cell homeostasis, lysosomal membrane integrity, and cell survival are all regulated by non-coding RNAs (ncRNAs) [72]. However, messenger RNA (mRNA) stability, RNA splicing, gene transcription, and protein degradation are all regulated by ncRNAs [72]. Specifically, ncRNAs control lysosome cell death and the transmembrane expression of specific lysosomal-associated proteins [72]. For instance, single-stranded non-coding RNA molecules called microRNAs (miRNAs), which have 21–23 nucleotides, are crucial for maintaining cellular homeostasis and regulating gene expression [73]. Therefore, aging-related illnesses such as CVDs, NDDs, and cancer are connected to miRNA dysregulation. The bioactive constituents of dietary components have the potential to control miRNAs [73]. Here, macronutrients, micronutrients, and trace minerals can modify the expression of certain miRNAs as potential approaches for delaying age-related health problems [73].

Rapamycin inhibits mTOR, a crucial kinase signaling pathway that coordinates information about energy sources, nutrient availability, and extracellular growth factor stimulation [74]. miRNA could interplay in mTOR response to several internal and external signals that touch numerous processes, including food intake, cellular metabolism, senescence, division, repair, and regeneration [73]. By promoting stress defense mechanisms, death/survival cascades, and decrease in inflammation, this increases a cell’s longevity. Thus, rapamycin, metformin, or resveratrol is used to postpone age-related disorders [74]. Moreover, the progress of anti-aging medications aided by the manipulation of transcriptomics, proteomics, lipidomics, metabolomics, microbiomes, and genomics [74]. Alongside that, caloric restriction, stem cell therapies, breaking AGEs, hormonal therapies, antioxidants, and telomere-based therapies are reported to increase cell longevity and lengthen the lifetime [74].

Interestingly, nanosystems can selectively release drugs in aged cells, cancer, or Alzheimer’s. Hence, nanostructures can drive medicines into the intracellular milieu for reducing or reversing cell senescence or aging [74]. Specifically, subcellular drug delivery systems could localize the medicines into the subcellular organelles in a specific manner [3]. Specifically target lysosomes play a role in regulating cellular responses to stress, death, repair, cell differentiation, and other processes [60]. Therefore, the restoration of lysosomal function is a promising lifespan-extending intervention [60]. For example, the administration of rapamycin and other lysosomal-acting medicines could act as essential regulators of cell homeostasis and delay age-related disorders [60].

### 2.6. Lysosomes in Cell Senescence

The exposure of the cells to harsh conditions either physical, chemical, or biological factors affects epigenetic, genetic, and proteomic processes. Thus, the cells undergo repair, necrosis, apoptosis, malignant transformation, or senescence. In this context, cellular senescence, also known as biological aging, is a highly significant cellular response that occurs in both physiological and pathological conditions. Cellular senescence is a conserved mechanism that pauses the growth of damaged cells [75]. Consequently, cellular senescence has a role in immunity, cancer prevention, tissue repair, and modeling. In contrast, a high number of senescent cells increases the risk of organ fibrosis, CVDs, NDDs, and other illnesses [75]. The cellular senescence is characterized by biomolecular damage, oncogenic activation, and telomere attrition. The release of the senescence-associated secretory phenotype (SASP) and permanent cell cycle stop are characteristics of cellular senescence [76,77]. Alongside cellular senescence, a number of cellular processes occur that are essential for aging, inflammation, and carcinogenesis [76].

Morphological changes, irreversible growth arrest, elevated lysosomal activity, induction of anti-proliferative proteins, resistance to apoptosis, and activation of damage-sensing signaling pathways are the most significant effects of cellular senescence [76]. Despite several studies linking lysosomal to cellular senescence, the exact machinery by which lysosomes contribute to this cellular phenomenon is still unclear [77]. Indeed, lysosomal alterations such as lysosomal enlargement increase in number, pH neutralization, permeabilization alteration, and accumulation of lipofuscin are demonstrated in senescent cells [77]. Moreover, the upregulation of lysosomal enzymes such as β-galactosidase [77]. According to a different study, senescence is linked to a rise in β-galactosidase activity, which is a biomarker of the senescent cells’ enlarged lysosomal mass [78]. Likewise, the lysosomal hydrolases such as α-mannosidase, α-fucosidase, and N-acetyl-β-hexosaminidase are markedly elevated in senescent cells [78]. Consequently, lysosomal senescence-associated hydrolase activity could be used as a biomarker for cellular senescence [22]. Besides, lipofuscins aggregate as lysosomal byproducts inside the senescent cells [22].

Additionally, the senescent cell secretome SASP is associated with cellular senescence [76,77]. SASP includes extracellular vesicles, metabolites, bioactive lipids, pro-inflammatory cytokines, chemokines, proteases, and mediators of inflammation [79]. Moreover, senescent cells secrete EVs that can act as signals affecting adjacent cells and induce an alteration in the cell’s phenotype characters [75]. The senescent cells are characterized imbalance between apoptotic/anti-apoptotic pathways, accordingly, they become immortal, despite the presence of damaged biomolecules and subcellular organelles [79].

## 3. Lysosomal Targeting as a Therapeutic Strategy

Lysosomes are the main subcellular organelles that deal with the breakdown of complex biomacromolecules, such as complex lipids, glycoproteins, proteoglycans, polysaccharides, and others, as well as damaged organelles and injured cells [80]. The resulting degradative products are engaging in biodegradative or biosynthetic pathways. Thus, lysosomes are essential for the recycling of damaged cellular constituents [80]. Also, lysosomes play a role in cellular defense mechanisms by aiding in the breakdown of foreign substances, infections, or poisons [80]. Because of the role of lysosomes in several cellular functions such as signal transduction, and other functions, these organelles are essential regulators of cellular homeostasis. Specifically, lysosomes are crucial for the processing of proteins, proteopathies, and organelle homeostasis [5,81]. Thus, lysosomes have broad lytic activities and are located at the midpoint of various biological processes such as cellular metabolism, nutrient sensing, ion homeostasis, and immunity [77]. Importantly, lysosomes have a vital role inside the cell as a housekeeping organelle and recycling of materials. Therefore, lysosomes are viewed as fundamental to critical cellular and organelle quality control to fight infection, protein aggregation, organelle damage, and oxidative stress [77]. Herein, lysosomes are members of regulators of cellular, growth, diversity, and death. However, lysosomal-aberration-induced aging, oncogenesis, autoimmune diseases, CVDs, NDDs, and other diseases [5,81]. Thus, lysosomes play a crucial role in the health and development of disease status. Nevertheless, lysosomal defect or overactivation is associated with numerous pathological conditions [5]. In this regard, although lysosome size raises encourage cell metabolism, they also weaken lysosomal membrane stability and increase the risk of cell expiry [5]. Therefore, the correction of lysosomal aberrations is a promising plan to prevent and cure ample diseases [5]. This could be achieved by combining genomics, transcriptomics, proteomics, and bioinformatics approaches [5].

In response to nutrient accessibility, stress resistance, apoptosis, plasma membrane repair, development, and cell differentiation, lysosomes are essential [60]. This pleiotropic is important for cellular and organismal survival and death. Thus, numerous age-related diseases and a decline in lifespan are linked to lysosomal abnormality [60]. Treatments that increase lifespan and promoting longevity are focusing on lysosomal quality [60]. Accordingly, the therapeutic window for the treatment of numerous pathological conditions may be opened by a thorough understanding of lysosomes as a double sword. Potential therapeutic targets in this context include modifying lysosomal acidification, lysosomal cathepsins, lysosomal biogenesis, and autophagy [5]. As well, restoration of lysosomal defects by small molecule therapy, macro-therapy, and lysosomal transplantation is a promising approach for controlling the disease’s dilemma. See Table 1.

**Table 1:** Examples of some FDA-approved lysosomal medications for the treatment of cancer, autoimmune disorders, and neurodegeneration by targeting lysosomes.

Disease	Medicine	Class/Mechanism	Specific Application
Cancer	Temsirolimus	Small-molecule therapy	Advanced renal cell carcinoma
	Everolimus	Small-molecule therapy	Advanced gastric cancer
	Chloroquine	Small-molecule therapy	Sensitizers with anticancer
	Brentuximab	Small-molecule therapy	Hodgkin lymphoma
Autoimmune disorders	Hydroxychloroquine	Small-molecule therapy	Systemic Lupus Erythematosus
Neurodegeneration	Milasen	Gene therapy	Neuronal Ceroid Lipofuscinosis

### 3.1. Manipulation of Lysosomal Signals

The targeting of lysosome acidification, calcium, enzymes, mTOR, biogenesis, and autophagy is promising for the treatment of lysosome-connected pathology [5]. Small-molecule therapeutics are involved in the manipulation of lysosomal signals (see Table 2).

#### 3.1.1. Manipulation of Lysosomal pH

The manipulation of the acidic media of lysosomal plays a vital role in different diseases. However, an acidic pH environment is the basis for the activity and functions of lysosomes [5,14]. Consequently, appropriate targeting strategies can be tailored according to the lysosomal acidification condition. For example, for cancer cells to sustain their high metabolic rate, an acidic environment is necessary. Likewise, a lysosomal acidic pH is involved in the overactivation of autoimmune cells to promote autoimmune diseases. On the contrary, impaired lysosomal acidification, proteopathies, and autophagy were observed in cellular senescence, aging, CVDs, and NDDs [5,81,82]. Thus, targeting lysosomal acidification could treat ample pathological conditions.

Medicines that inhibit lysosomal acidification include v-ATPase inhibitors and antimalarial drugs. v-ATPase inhibitors act by pumping protons into the lysosome or late endosome [5,83]. Inhibitors of v-ATPase include bafilomycin A1, concanamycin, archazolid A, and other drugs [5,84]. Moreover, bafilomycin A1 can prevent autophagosome–lysosome fusion by focusing on lysosomal acidification-inducing endoplasmic reticulum calcium pump [5,85]. Moreover, salicylihalamide A inhibits lysosomal function by interrupting v-ATPase activity [5,84]. The next generation of v-ATPase inhibitors, such as NiK12192 and other substances, is designated in a number of studies [5,84].

Antimalarial medicines are the sole class of autophagy inhibitors utilized in therapeutic settings [5,86]. For instance, after protonation, chloroquine and hydroxychloroquine can accumulate in lysosomes due to their shared cationic amine group structure, which results in lysosomal deacidification [5,86]. Additionally, chloroquine and hydroxychloroquine can prevent autophagosomes from fusing with lysosomes, which inhibits autophagy [5,86].

The drawbacks of chloroquine and its derivative are ocular toxicity and retinopathy at high doses in long-term use [5,86]. Quinacrine is another antimalarial medication that is 60 times more effective than chloroquine at lysosomal deacidification and would be a better option for autophagy inhibition [5,38]. Compared to other medications, antimalarial derivatives exhibit strong lysosomal localization and autophagy inhibition properties [5,86]. Moreover, by interfering with the lysosomal localization of v-ATPase subunits, palmitoyl-protein thioesterase-1, which shared monomeric and dimeric chloroquine derivatives, caused dramatic lysosomal deacidification [5,87]. Palmitoyl-protein thioesterase 1 causes retinopathy such as maculopathy with pigmentary [5,88].

By focusing on chemicals that inhibit lysosomal acidification, it is possible to restore it in cells with compromised autophagy [5,89]. Additionally, targeting mutated palmitoyl-protein thioesterase 1 partially restores lysosomal acidity and autophagy. Moreover, in three distinct NDD models, acidic nanoparticles were internalized into lysosomes and repaired the faulty autophagy–lysosomal and lysosomal acidification pathways [89].

#### 3.1.2. Manipulation of Lysosomal Cathepsins

Lysosomal cathepsins are among the functional secretomes of lysosomes [5,14]. Different cathepsins are well-studied as they have roles in the pathophysiology and therapy of several diseases [5,45]. By breaking down the extracellular matrix, cathepsin B and cathepsin L secretion mediate melanoma metastasis [5,90]. Thus, lysosomal cathepsins and other lysosomal enzymes are crucial for angiogenesis, invasion, proliferation, and resistance to anticancer drugs [5]. Moreover, lysosomal cathepsin expression is usually upregulated in leukemia, melanoma, breast cancer, and gastrointestinal cancer [5]. Likewise, deregulated cathepsins are essential for the development of NDDs and autoimmune diseases. Thus, it was proposed that cathepsins would be an excellent target for the treatment of autoimmune disorders, cancer, and NDDs [5,91].

Several medications showed better specificity and efficiency than cathepsin inhibitors [5,92]. For example, odanacatib and other cathepsin K inhibitors are thought to be the most promising options for treating cancer-induced bone loss [5,93]. Likewise, targeting cathepsin secretion or cathepsin antibodies may be a viable therapeutic approach to address the aberrant activity of lysosomal cathepsins [5,14]. Due to cathepsin D’s protective function in cardiac remodeling, exogenous supplementation or induction of expression of cathepsin D may be beneficial for the relief of cardiac diseases [5].

A class of recessive lysosomal diseases known as neuronal ceroid lipofuscinosis is characterized by compromised lysosome-autophagy pathways [5,94]. The injection of the viral vector encoding mouse cathepsin D into the cerebrum showed an increase in the lifespan of cathepsin D-knockout mice [5,94]. Moreover, mice given recombinant human procathepsin D had improved autophagic flux and reduced lysosomal storage accumulation in the viscera and central nervous system [5,95].

The viability of restoring lysosomal cathepsins in illnesses linked to decreased cathepsin efficiency is supported by these studies. Targeting cathepsins for clinical application is challenging due to the lack of knowledge and complexity of cathepsin function [5].

#### 3.1.3. Manipulation of Lysosomal Membrane

Stress causes lysosomes to burst, and the contents of these ruptures may cause inflammation and cell death when they leak into the cytosol [5,26]. Because lysosomes lack antioxidant enzymes like superoxide dismutase, their membrane is more vulnerable to damage during free radicals attack [5,96]. Therefore, the manipulation of lysosomal membrane integrity can be exploited as therapeutic opportunities [5,26]. Free radicals are produced as a byproduct of conventional anticancer medications, but they can also be brought on by photodynamic therapy or iron exposure [5,97]. Artemisinin’s lysosomal release of iron can cause malignant cells to produce free radicals and permeabilize their lysosomal membranes [5,97].

Additionally, direct disruption of the lysosomal membrane protein by agents such as mycotoxin enniatin B1 can also induce lysosomal membrane permeabilization [5,98]. Also, by targeting acid sphingomyelinase, heat shock protein 70 (Hsp70) can cause sphingomyelin accumulation, which can in turn cause lysosomal membrane permeabilization [5,26]. Acid sphingomyelinase was effectively inhibited by direct inhibitors like zoledronic acid and cationic amphiphilic medications, which also cause a loss of lysosomal membrane integrity [5,26]. Antimalarials, antidepressants, and antihistamines are examples of clinically used cationic amphiphilic medications that can cross lysosomal membranes and build up inside the lysosomes following protonation [5,26]. Furthermore, terfenadine and amitriptyline are cationic amphiphilic drugs that showed the inhibition of acid sphingomyelinase and induce lysosomal membrane permeabilization in targeted cells [5,99]. Medicines such as quercetin, triptolide, etoposide, and others can induce lysosomal membrane permeabilization and subsequent cell death [5,26]. These agents can induce serious adverse reactions due to the simultaneous inhibition of other organelles HSP70 [5,100].

Indeed, multiple defense mechanisms can be developed by cells to prevent lysosomal rupture and subsequent cell death [5,101]. The endosomal sorting complex required for transport (ESCRT) machinery can repair the limited permeabilization of the lysosomal membrane. Lysophagy machinery, a selective autophagy process brought on by the ubiquitination of lysosomal proteins, can remove damaged lysosomes [5,101]. ESCRT machinery and its component recruitment were found to depend on calcium outflowing from lysosomes [5,102].

The ubiquitination used for autophagy increases the exposure of lysosomal glycans and induce directly the ubiquitination of damaged lysosomal proteins [5,102]. For example, instead of using the ESCRT machinery, myoferlin stabilizes the lipid bilayer or encourages the fusion of lysosomes with other vesicles that act as membrane donors [5,103]. This action provided early-acting protection against membrane damage. On the contrary, Knocking out myoferlin mediates lysosomal dysfunction and reduces tumor growth [5,103].

Likewise, inducing mitochondrial membrane permeabilization and targeting the microtubule cytoskeleton were found to mediate lysosomal membrane permeabilization and cell death [5,26]. Furthermore, microtubule inhibitors such as taxenes and vincristine can mediate lysosomal membrane permeabilization [5,26]. Additionally, crosstalk between autophagy and lysosomal membrane permeabilization would provide more options for targeting lysosomal membrane permeabilization. For example, trehalose is an effective autophagy inducer and acts by inducing lysosomal enlargement and lysosomal membrane permeabilization. Also, knocking down certain autophagy proteins induced lysosomal membrane permeabilization and apoptosis [5,104].

#### 3.1.4. Manipulation of Lysosomal Calcium

Lysosomal calcium elicits a significant role in lysosomal biogenesis, acidification conservation, and reorganization. Moreover, vesicle trafficking such as autophagy and endocytosis are lysosomal calcium-dependent machinery [5,14]. Transient receptor potential mucolipin channels (TRPMLs) and two-pore channels are two types of calcium channels that are suitable targets because of their location on the membranes of the endo-lysosomal system [5,14]. Mucolipin 1-3 encodes each of the six transmembrane domain channels known as TRPMLs (TRPML1-3) [5,105]. The most studied channel is TRPML1, which is connected to lysosome biogenesis and various membrane fusion processes [5,106].

TRPML1 was upregulated as a tool for drug resistance in certain cancer types, including melanoma, bladder urothelial carcinoma, and triple-negative breast cancer [5,107]. However, some cancers such as non-small-cell lung carcinoma and glioblastoma showed downregulation of TRPML1 and cytotoxicity can be induced by TRPML1 agonists [5,107]. Thus, TRPML3 was linked to the course, aggressiveness, and prognosis of pancreatic ductal adenocarcinoma, whereas TRPML2 was involved in chemokine trafficking and secretion in murine macrophages [5,108].

The heterogeneous expression of calcium channels in malignant cells and the lack of specific targeting agents raise difficulties in targeting lysosomal calcium signaling for cancer therapy [5]. Some non-selective modulators of calcium channels have been synthesized to target phosphoinositide cascade and showed the inhibition of autophagy-dependent cancer cells [5,109].

In the endo-lysosomal system, two-pore channels are voltage-gated ion channels that use the calcium-mobilizing messenger nicotinic acid adenine dinucleotide phosphate to trigger calcium signals [5]. Compounds that target nicotinic acid adenine dinucleotide phosphate such as tetrandrine and others showed great activity in reducing the migration and adhesion of malignant cells [5,106]. Similarly, there is an important role of lysosomal calcium in NDDs; thus, compounds that correct the calcium aberration in lysosomes are promising in the treatment of NDDs and other diseases [5,110].

#### 3.1.5. Manipulation of mTOR

Information about extracellular growth factor stimulation, nutrient availability, and energy sources is integrated by mTOR, a kinase at key signaling [74]. The appropriate recruitment, assembly, and activation of mTOR signaling elements are carried out by the lysosomes [5,111]. Accordingly, lysosomes and mTOR form a connected metabolic cascade [5,111]. The lysosomal injury cause deterioration of mTOR cell signaling [5,111]. Frequently mTOR signaling modulates cell metabolism, proliferation, and malignant transformation. Therefore, the targeting of mTOR can be utilized as therapy for age-related consequences, malignancy, and several pathological conditions [5,111]. Moreover, manipulation of mTOR affects the lysosomal biogenesis and autophagy machinery. Thus, the mTOR modulators can be utilized as therapy for autophagy-related diseases such as aging, NDDs, and other diseases [5,81,112]. Rapamycin and its analogues, such as temsirolimus, evero-limus, and ridaforolimus, are mTORC1 inhibitors [5,113]. Other compounds such as AZD2014, CC-223, and TAK-228 are documented to inhibit mTORC1 and mTORC2 [5,113].

**Figure 4:**
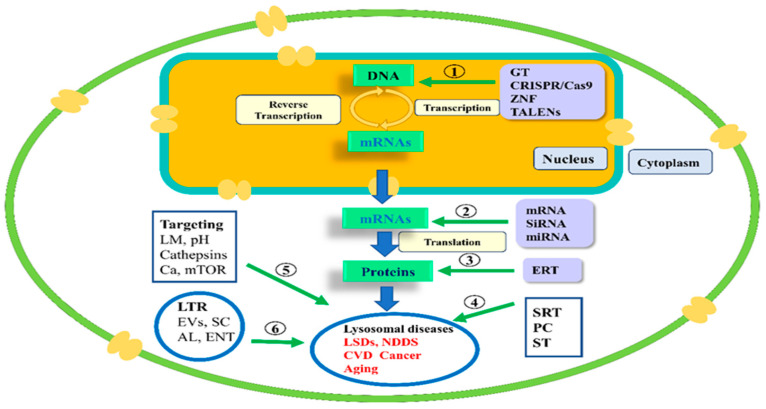
Therapeutic modalities of lysosome-related diseases. 1, Lysosomal medications acting on DNA, 2, Lysosomal medications acting on mRNA, 3, Lysosomal protein medications, 4, Substrate reduction, pharmacological chaperons, and Senotherapeutics, 5, Medication targeting lysosomal membrane, pH cathepsins, calcium and mTOR, 6, Lysosome transplantation (LTR), extracellular vehicles (EVs), stem cells (SC), artificial lysosome (AL), endosymbiotic therapy (ENT).

Additionally, mTOR1 and mTORC2 signals are regulated by phosphatidylinositol 3-kinase (PI3K) or serine/threonine kinase 1 (AKT), inhibiting both PI3K and AKT at the same time can block both mTORC1 and mTORC2 [5,113]. Therefore, PI3K and AKT are potential targets for the development of anticancer agents such as buparlisib, pictilisib, ipatasertib, and capivasertib [5,114]. Moreover, the guanine nucleotide exchange factor for Ras-related GTP-binding protein GTPases recruits mTORC1. In this regard, knocking out the guanine nucleotide exchange factor attenuates aberrant mTORC1 activation [5,115]. Additionally, substances like NR1 have the ability to strongly and specifically block mTORC1 signaling [5,116]. Furthermore, Nemo-like kinase is a subfamily of MAP kinase that can phosphorylate mTORC1 components, inhibiting mTORC1’s lysosomal localization [5,117]. Despite this, the toxicity of the long-term application of mTOR inhibitors is not fully addressed [5,114]. Figure 4 displays the therapeutic modalities of LDs.

### 3.2. Restoration of Lysosomal Defects

Several therapeutic modalities are proposed to restore the lysosomal defects, including small-molecule therapeutics, ERT, gene therapy and lysosomal transplantation. Table 3 indicates some of clinically used medication for LSD management.

#### 3.2.1. Small-Molecule Therapeutics

The use of small-molecule drug approaches overcomes the limitations of macromolecule lysosomal therapeutics that cure LDs [118]. Mechanistically, proteostasis regulators, pharmacological chaperone therapy, and SRT are all included in small molecule therapy [118]. SRT reduces the production of glycosphingolipids and other complex biomolecules in the lysosome [118]. Miglustat, for instance, is a medication that is approved to treat lysosomal diseases such as Gaucher disease and Niemann Pick disease type C [118]. Miglustat is a competitive and reversible inhibitor of the enzyme glucosylceramide synthase. Consequently, Miglustat stops the formation of the enzyme substrates, here, the enzyme doesn’t work [119]. Chaperone therapy increases the amount of an active lysosomal enzyme by dictating the correct folding of lysosomal enzymes [118]. In Fabry patients with missense mutations, migalastat is documented as therapeutic option. Migalastat is an alpha-galactosidase A chaperone that is used for Fabry disease in patients with an amenable galactosidase alpha gene variant [120]. Moreover, ambroxol is a medication primarily used as a mucolytic agent by thinning mucus. In the last years, ambroxol was indicated as a hopeful chaperone therapy for Gaucher disease, however, ambroxol selectively binds to misfolded glucocerebrosidase, the enzyme deficient in Gaucher disease [121,122]. This binding corrects the folding and trafficking of glucocerebrosidase to lysosomes for glucocerebroside and glucosyl sphingosine degradation. However, the accumulation of these materials induced Gaucher disease [121,122]. Moreover, ambroxol can cross the blood–brain barrier, compared with traditional enzyme replacement therapies [121,122]. In this context, several studies documented ambroxol for Gaucher disease management [121]. Additionally, ambroxol appears to enhance mitochondrial function in cells from those with neuronopathic Gaucher disease, suggesting broader cellular benefits beyond just improving glucocerebrosidase activity [121]. Thus, ambroxol elicits notable improvements in neurological symptoms such as myoclonus and seizures [121,122]. In this regard, ambroxol represents a novel and effective avenue for treating neuronopathic Gaucher disease [121,122].

Small molecules in proteostasis regulators cause lysosomal enzymes to misfold by influencing the body’s natural chaperone system and signaling pathways, including calcium channel-signaling pathways [118]. The off-target actions that cause drug toxicity are the main obstacles to small molecule therapy. The fabrication of peptides, polymers, and nanocarriers are proposed expand the effectiveness of small-molecule–lysosomal therapy with improved therapeutic index [9,10].

#### 3.2.2. Macromolecule Therapeutics for LSDs

LSDs represent a group of inherited metabolic disorders that can have profound impacts on children [33,123]. These conditions arise from deficiencies in specific lysosomal enzymes, resulting in the harmful accumulation of substrates within lysosomes [33,123]. For example, Gaucher disease is caused by a deficiency in glucocerebrosidase, leading to the buildup of glucocerebroside [33]. Affected children have hepatosplenomegaly, anemia, bone pain, and growth delays, with neurological issues [33]. The standard treatment involves intravenous administration of glucocerebrosidase, such as imiglucerase [33]. Fabry disease, another LSD, results from a deficiency in α-galactosidase-A, causing the accumulation of globotriaosylceramide [33,123]. In this case, children may suffer from painful crises, skin lesions, and gastrointestinal problems, and renal problems. Treatment options like agalsidase-beta or agalsidase-alpha help manage symptoms and prevent complications [33,123]. Pompe disease is another LDS linked to a deficiency in acid alpha-glucosidase, leading to glycogen accumulation [33,123]. In its infantile form, symptoms typically include hypotonia, progressive weakness, cardiomyopathy, and respiratory difficulties. Treatment with alglucosidase-alpha enhances muscle strength and respiratory function [33,123].

Mucopolysaccharidosis (MPS) types I, II, and VI arise from deficiencies in enzymes responsible for breaking down glycosaminoglycans [33,123]. Children with MPS may face skeletal abnormalities, growth delays, joint stiffness, and varying degrees of cognitive impairment [33]. ERT such as laronidase for MPS-I, idursulfase for MPS-II, and arylsulfatase B for MPS-VI are employed to manage symptoms and enhance quality of life. Krabbe disease, caused by a deficiency in galactocerebrosidase, leads to the accumulation of psychosine [33,123]. Symptoms of this disease usually manifest in infancy, including irritability, developmental delays, and spasticity [33,123]. Additionally, lysosomal acid lipase deficiency (LAL-D), a member of LSDs caused by deficiencies in specific lysosomal enzymes, leads to the accumulation of substrates within lysosomes [33,123]. LAL-D specifically results from a deficiency in lysosomal acid lipase, causing triglycerides and cholesteryl esters to accumulation resulted as dyslipidemia, hepatosplenomegaly, growth delays, and hepatic dysfunction [33,123]. Therapeutic approaches for managing LAL-D include ERT with sebelipase-alfa reduce liver fat content, and improve liver function [33,123]. Moreover, gene therapy aims to correct the underlying genetic defect in LSD children [33,123]. SRT decreases substrate synthesis as well as PC stabilize misfolded enzymes [33,123], and provide supportive care to manage symptoms and complications [33,123].

Niemann-Pick disease (NPD) encompasses a group of rare genetic disorders marked by the harmful accumulation of lipids, especially sphingomyelin, due to enzyme deficiencies. The most prevalent forms are NPD types A and B, both resulting from mutations in the SMPD1 gene, which encodes the viral enzyme acid sphingomyelinase [124,125]. This enzyme plays a critical role in breaking down sphingomyelin, a type of sphingolipid, leading to its buildup in crucial organs like the liver, spleen, and brain [124,125]. NPD-A is the most severe variant, typically diagnosed in infancy, presenting with symptoms such as hepatosplenomegaly, neurological decline, and a distinctive cherry-red spot in the eye. Tragically, most children affected by this type do not survive beyond three years due to profound neurological impairment [124,125]. In contrast, NPD-B has a later onset and is characterized by hepatosplenomegaly without significant neurological issues, allowing patients to reach adulthood, though they may face progressive liver and lung complications [124,125]. NPC, not directly linked to sphingomyelin metabolism, involves cholesterol transport defects caused by mutations in the NPC1 and NPC2 genes, leading to symptoms that can manifest at any age, including neurological decline and organomegaly [124,125]. In NPC, there is an increase in sphingosine storage leads to calcium increased concentration that blocks autophagy and increases the accumulation of cholesterol, sphingomyelin, and glycosphingolipids [33]. Current treatment strategies for NPD focus on symptom management and slowing disease progression. ERT is primarily employed for NPB, delivering the missing enzyme to help reduce lipid buildup [124,125]. Additionally, SRT aims to lower the synthesis of sphingomyelin and other accumulating substrates, thereby mitigating their levels in the body [124,125]. Promising research is underway to explore gene therapy as a potential corrective measure for the underlying genetic defects associated with NPD [33,124,125,126]. LSDs therapy still challenges in pediatric populations. Early diagnosis and intervention are essential for maximizing the benefits of these innovative therapies [33,123]. Early intervention through hematopoietic stem cell transplantation can be beneficial, potentially improving outcomes [33,123]. Supportive care encompassing physical therapy, nutritional support, and management of complications of LSDs [124,125]. Monitoring of liver function, and addressing growth delays are crucial for improving the quality of life in LSDs affected children [33]. Advancements in therapeutic approaches particularly ERT and stem cell transplantation offer hope for better management and outcomes of LSD patients [33,123]. Ongoing research continues to explore innovative treatment options for LSDs [33,123].

##### Enzyme Replacement Therapies (ERTs)

ERT is the first commercial biological therapy used to treat lysosomal diseases [118]. One effective therapeutic approach for the management of LDs is enzyme replacement therapy. In this approach, the enzyme cargoes are delivered into the lysosomes utilizing the M-6-P ligand that is imported into the lysosomes by M-6-P receptors [118]. Upon the successful delivery of the cargoes, the enzymes are liberated into the lysosomal milieu to degrade the accumulated substrates and alleviate LD symptoms [118]. Recombinant enzyme replacement therapy is used to treat a number of LDs, including mucopolysaccharidosis, Fabry disease, Gaucher disease, and Pompe disease. Moreover, many enzymes are under clinical trials for the treatment of LDs [118]. Despite ERT being crucial for treating MPS and other LSDs, it faces several limitations [127,128]. ERT has limited capacity to cross biological barriers like the blood-brain barrier and others biological barriers [118]. The ERT is rapidly cleared from the blood with short plasma half. This limits their therapeutic window and requires frequent infusions [127,128]. Moreover, patients may develop immunological reactions and produce antibodies against the infused enzyme, reducing treatment efficiency and causing side effects [127,128]. Several approaches were proposed to overcome the limitation of ERT enhancements such as developing long-circulating enzymes for less frequent dosing, bone-targeting enzymes to better address skeletal manifestations, and alternative production methods to reduce costs and improve availability [127,128]. There is also growing interest in the production of recombinant enzymes and combination therapies, such as pairing ERT with gene therapy or SRT, to improve overall efficacy [129]. The PEGylated enzymes and high-dose formulations are suggested to enhance the pharmacokinetics and pharmacodynamics of ERT [129]. The future of ERT lies in innovative therapies, improved delivery mechanisms, and personalized medicine, with ongoing clinical trials necessary to validate these approaches [129].

Several studies were focused on enzyme modification and delivery approaches using nanocarriers for efficient enzyme delivery. The fabrication of lysosomal enzymes in nanocargoes can overcome the problems of enzyme replacement therapies such as immunologic reactions and biodegradation [27]. Moreover, enzyme nanocargoes limit non-selective biodistribution and improve the biological response, however, nanodevices such as nanoparticles, and other proteins increase drug absorption extend enzyme release, and improve pharmacokinetics parameters [27].

The liposomal strategy was reported as delivering cargo for Fabry disease and Batten disease. In the last years, EVs have been proposed as biological cargo for enzyme replacement therapy delivery for Gaucher disease and other LDs [27]. Alongside, conferring enzyme protection by EVs, they permit enzymes ability to overwhelm the biological barriers such as the BBB and other barriers to access the site of action [27]. Additionally, a promising approach to treating inherited diseases, LDs, and age-related disorders is the advancement of nucleic acid therapy and gene editing technology [9,10]. EVs are promising delivery vehicles for targeting proteins, nucleic acid therapy, and gene editing technology [9,10].

##### Gene Therapies

Since LDs, whether inherited or acquired, frequently impact the central nervous system, research into central nervous system-targeting therapies is encouraged [130,131]. For LDs that involve the central nervous system, there are two methods of gene delivery. In one scenario, the gene carrier is given in vivo, either by intravenous delivery of the genetic cargoes that are specific to central nervous systems or by in situ administration of the faulty genes [130,132]. For LDs that affected organs rather than central nervous systems, the gene delivery cargoes could target the organs using a specified carrier [130,133].

Gene editing techniques for the treatment of lysosomal dysfunctions are delivered with the help of nanobiotechnology approaches. Extracellular vesicles, like exosomes, can be used in these situations to deliver gene editing techniques for the treatment of mucopolysaccharidoses [80]. It has been documented that, there is a clinical trial study in children this study, the gene delivery for treatment LDs achieved by intraparenchymal injections of a recombinant viral vector in vivo [130,134].

Furthermore, since microglial cells develop from the differentiation of hematopoietic precursor cells, another strategy is to use hematopoietic stem cell-targeted gene delivery [130]. Here, the patient receives an infusion of the hematopoietic stem cells after they have been extracted and corrected ex vivo. Repopulating the bone marrow, gene-corrected hematopoietic stem cells move into the central nervous system and develop into microglial cells [130]. Enzymes, healthy neurons, and other brain cells are then secreted by the microglial cells. An ex vivo gene delivery procedure was used on eight of the nine children who had metachromatic leukodystrophy [130]. They witnessed the reduction of disease progression or the prevention of disease onset, demonstrating the potential of this strategy [130].

Preclinical trials for various LDs examined genome editing using clustered regularly interspaced short palindromic repeats linked to caspase-9 (CRISPR-Cas9) [135]. In a Hunter syndrome trial, only zinc finger-mediated gene editing was used [136]. Additionally, by regulating gene expression or focusing on cellular RNA through a variety of methods, antisense oligonucleotide technology is a potent therapeutic alternative for the treatment of genetic disorders [130]. Single-stranded DNA could be chemically modified to create more effective next-generation antisense oligonucleotides [130]. For the treatment of Duchenne muscular dystrophy and spinal muscular atrophy, antisense oligonucleotide therapies were authorized [130]. The primary goal of antisense oligonucleotide treatments for LDs is to get mutated transcripts to splice normally again. Patients with LDs have been found to have at least 600 mutations that impact the splicing of precursor mRNA. Up to 70% of the pathogenic alleles for Pompe disease, Fabry disease, mucolipidosis type II/III, and Tay-Sachs disease are caused by a common splicing variant, which makes it a prime candidate for therapeutic gene editing technology [130]. A cellular model of nuclear pore complexes with a disease-carrying variant was used to test antisense oligonucleotide therapy for LDs, and the approach was successful in restoring normal splicing [130]. Several other studies showed that gene editing approaches in the treatment of certain types of LDs [130]. In this context, Kim et al. [137] revealed the creation of milasen, a splice-modulating antisense oligonucleotide medication designed specifically for a single patient suffering from a neurodegenerative disease that is fatal [137]. In such cases, there is a misplacing of certain mRNA resulting in premature translational termination. Therefore, the antisense oligonucleotide was designed to increase the ratio of normal to mutant mRNA [137].

The Food and Drug Administration (FDA) authorized the intrathecal injection of Milasen in increasing dosages for this individual patient [137]. Compared to 30 seizures per day lasting one to two minutes prior to treatment, this therapy demonstrated that the seizures decreased to just a few episodes lasting a few seconds. Less than a year passed between the discovery of the variations and the clinical use of customized medications [137]. About 23 orphan medications have been authorized to treat 11 lysosomal disorders. Four diseases—Gaucher disease (six medications), cystinosis (five medications), Pompe disease (three medications), and Fabry disease (two medications)—have multiple approved treatments. The FDA only approved one medication for each of the other seven diseases [138]. Also, the great results of lysosome-related preclinical research increase the interest in the clinical application of targeting lysosomes in cancer and other lysosomal-associated diseases [5].

#### 3.2.3. Senotherapeutics

The altered pathways in senescent cells are potential targets for the design of senolytic and senomorphic agents as senotherapeutic medicines. Medicines that encourage selective cell death of senescent cells are named senolytic agents [79]. Others that suppress the markers of senescence such as SASP, are called senomorphic agents. Accordingly, the development of senolytic and senomorphic agents could delay age-related diseases and extend the cell’s longevity [79]. In this context, when senescent cells are removed through genetic or pharmaceutical means, aging-related illnesses like cancer, NDDs, CVDs, and numerous other conditions are delayed in their onset [76].

The abundance of lysosomes in senescent cells might push autophagy, thus, the triggering of autophagy by therapeutic interventions could induce senescent cell death. However, autophagy causes activation of cell death pathways inhibited within senescent cells [22]. In this context, senolytic agents can increase lysosomal enzyme activity, this can be achieved by prodrugs that are activated by such as senescence-associated beta-galactosidase. It has been observed metformin, fibrate, and mTORC1 inhibitors enhanced lysosomal activity [22].

Additionally, the senescent cells’ altered membrane potentials and proton concentrations are exploited by the cardiac glycosides [22]. Moreover, when senescent cells resist the lowered pH caused by lysosomal changes and by upregulating buffering systems like the glutaminase product ammonia, compensatory responses to the chemical changes can be targeted. Drugs can target the enzymes that maintain buffer concentrations, and altering these buffering systems would promote senescent cell death [22]. Moreover, senolytic agents can target key proteins involved in apoptosis including B-cell lymphoma-2 (BCL-2), and other antiapoptotic proteins [76]. Here, senolytic medicines include dasatinib, BCL-2 inhibitors, forkhead box protein O4-cellular tumor antigen-53 (FOXO4-p53) inhibitors, HSP90 inhibitors, and ubiquitin-specific-processing protease 7 (USP7) inhibitors. Natural products and their analogs such as polyphenols, cardiac glycosides, piperlongumine, quercetin, fisetin, curcumin, statins, galactose-modified senolytic pro-drugs and other natural products [79,139].

Senomorphics target a variety of pathways, such as p38, mTOR, PI3K/AKT, nuclear factor kappa light chain enhancer of activated B cells (NF-κB), mitogen-activated protein kinase (MAPK), and other cascades, to inhibit SASP functions [76]. Consequently, senomorphics medicines suppress the detrimental effects of SASP. The senomorphics include rapamycin, metformin, resveratrol, and aspirin [79]. Moreover, immunotherapy using monoclonal antibodies against individual SASP components could fight cell senescence [76]. However, numerous secretions of growth factors, pro-inflammatory cytokines, and other cell signaling molecules are indicative of cell senescence [140].

Indeed, many signaling pathways, such as NF-κB, JAK/STAT, mTOR, and other cellular cascades, start SASP [140]. An immunosuppressive tumor microenvironment is induced and cancer progression is deteriorated by abnormal stimulation of the Janus kinase 2/signal transducer and activator of transcription 3 (JAK2/STAT3) signaling pathway. Thus, focusing on SASP is a viable approach to finding anticancer drugs [140]. Redox imbalance has been shown in multiple studies to cause lysosomal dysfunction and cell senescence. To restore lysosomal function, therapeutic approaches based on redox balance regulation are used [32]. The goal of the nanomedicines is to deliver senotherapeutics. Interestingly, nanomedicines can accumulate in lysosomes after being internalized by tumor and senescent cells through endocytosis [32]. Also, the loading of medicines into galactose-polymer-coated nanocargoes can be specifically released into the lysosomes of senescent cells [22]. In non-senescent cells with high senescence-associated beta-galactosidase activity, like macrophages, these tactics have poor specificity effects [22].

Despite nanoparticles eliciting marked improvement in therapeutic impact in experimental studies, their clinical uses are still limited and challengeable [32]. When combined, treatment approaches based on controlling the disruption or restoration of lysosomal function help to identify new approaches for treating a range of diseases [32].

#### 3.2.4. Endosymbiosis Therapy

The endosymbiotic concept is recognized as prototypical for the beginning of mitochondria and chloroplasts the bacteria [29]. Directed endosymbiosis can be useful in biotechnology, cell compartmentalization, and medicine [29]. Specifically, endosymbiosis could be harnessed in organelle transplantation and the production of artificial organelles for medical applications in the treatment of organelle-related diseases [29]. For instance, mitochondria are essential for apoptosis, cell cycle control, and energy production [29]. The disruption or deregulation of mitochondrial function leads to diabetes, cancer, diabetes, muscular degeneration NDDs, and cellular aging [29]. Thus, the mitochondria or other subcellular organelles could be transplanted for the restoration of normal cellular function [141]. Additionally, the cell fusion approach could be harnessed for organelles transplantation, however, it is a form of intercellular communication. In this approach, the subcellular organelles could be permanently exchanged between the fused cells [142]. It was reported that the transplant of organelles restored the survival and function of cardiomyocytes. This is accomplished by the damaged heart cells’ continuous transmission of stem cell mitochondria [142]. Numerous studies on the transfer of intercellular organelles between distinct stem cell types and differentiated cells in a range of disease models, such as respiratory, cardiovascular, ocular, kidney, cancer, diabetes, and others, are currently available [142].

Both homotypic (mesenchymal stem cell to mesenchymal stem cell) and heterotypic (mesenchymal stem cell to cardiomyocyte) organelle transplantation may be performed between cells from the same species and those from different species [143]. In this context, the concurrent transmission of multiple types of organelles was reported, and other literature documented the transfer of a single type of organelle between two specific cell types [143]. Here, the list of cellular organelles and cell types that are known to engage in organelle transfer has been established [143]. Additionally, nanotubes can act as pathways for the transfer of intracellular organelles [143]. It is common for the lysosomes and mitochondria to move from the uninjured cell to the injured one with directional specificity [143]. However, the donor organelle is the one that shoots the nanotube in a pair of cells connected by them, and the recipient cell is the other one [143].

The quickly developing field of creating new biological systems is known as synthetic biology. The development of genome editing technology has enabled this highly interdisciplinary field [29]. The engineering of new endosymbiotic relationships between two or more species is an area that has not been thoroughly studied [29]. Collectively, the use of endosymbiosis and chemical means for the production of artificial organelles can restore cell function and improve or prevent disease by organelle replacement [29,144]. However, subcellular organelles achieve specific roles and their dysfunction can lead to developmental defects, aging, and ample disease conditions [144].

The fully produced artificial organelle with membrane structure and catalytic function could be manufactured and implanted within the intracellular milieu to re-establish the cellular function. These approaches are bioinspired by endosymbiosis theory [141]. It is feasible to create more optimized genetic networks to enhance cellular functions that take place in organelles, such as oxidative phosphorylation, respiration, or other organelle processes, by comprehending the biochemical makeup and metabolic functions of an organelle [29]. Moreover, the improvements in traits such as the increased growth rate and biomass, of microbe and plant species could be exploited in bio-industrial applications [29].

The organelles are separated from the rest of the cell, but directed endosymbiosis can also be used to address biotechnologically significant problems like cellular containment or adjusting environmental conditions [29]. Similarly, diseases involving mitochondria could be studied using artificial organelles as models. Essentially, patients’ damaged mitochondria could be replaced or repopulated using engineered organelles as a therapeutic intervention [29]. One promising method for creating next-generation targeting strategies for the diagnosis and treatment of diseases at the organelle level is the feasibility of endosymbiosis and chemical methods for the creation of artificial organelles [29,144].

##### Lysosomal Transplantation

Intracellular milieu is organized into subcellular organelles to perform specific functions. Thus, pathophysiological conditions such as developmental defects, autoimmune disease, aging, cancer, CVD, NDDs, diabetes, and other diseases can result from the malfunction of subcellular organelles [144]. Thus, organelle transplantation is a promising tactic to reestablish the organelle’s job as a method to prevent or cure diseases [144]. Here, organelle transplantation is not only a strategy for the diagnosis but also could treat the diseases at the organelle level [144].

Despite the organelle transference that exists for mitochondria, the transplantation of ribosomes and lysosomes has been reported [141]. As well, scientists have also been interested in producing synthetic analogs of organelles [141]. The goal of creating artificial subcellular organelles is to create catalytically active compartments that can be integrated with living cells by designing enzyme-loaded nanosized vesicles [141]. This integration could restore cellular function and correct malfunctioning processes [141]. Numerous studies have indicated that lysosomes are transported between cells using EVs, cellular fusion, and other methods [142].

EVs such as exosomes, microvesicles, and apoptotic bodies are promising choices for organelle transplantation [142]. However, EVs mediate the crosstalk between the cells, upon liberation by donor cells, they are subsequently uptake by acceptor cells [145]. The cargoes of EVs are biomolecules such as lipids, nucleic acids, proteins, enzymes, or subcellular organelles. Thus, EVs could be used as macro therapy and organelle therapy [145]. Interestingly, EVs have the endosomal escaping ability, consequently, the cargo biomolecules and organelles a evading the lysosomal degradation [145]. EVs can be harnessed to transfer mitochondria, ribosomes, polyribosomes, and lysosomes [142]. It has been established that lysosome transmission between the cells improves cell survival under stress conditions [143]. In this sense, glycated collagen type I exposure impairs autophagy, raises lysosomal pH, and causes lysosomal dysfunction, all of which decrease the survival of human umbilical vein endothelial cells [146]. Improved cell survival results from the transplantation of lysosomes from the endothelial progenitor cell to the human umbilical vein endothelial cell [146]. However, the formation of nanotubes is inhibited, lysosomal transfer is delayed and the cell’s survival is decreased [146].

Despite intercellular organelle transmission might cause pathophysiology, the majority of studies on this theme focus on the helpful impact of organelles transplantation [143]. In vitro, macrophages sprout nanotubes to mediate cellular crosstalk endosomes and lysosome relocation [147]. These endosomes could transfer from donor cells to recipient cells [147]. Indeed, the physiological process of melanosome transfer from the melanocyte to the keratinocyte, which regulates skin and hair pigmentation, is accelerated by stressors like UV exposure [148]. In the culture of mouse endothelial progenitor cells and human umbilical vein endothelial cells in standard media, the human endothelial cells receive mitochondria from the endothelial progenitor cells [149]. Treatment of this culture media with adriamycin enhanced the organelles transplantation [149]. Human umbilical vein endothelial cells receive mitochondria from endothelial progenitor cells in a unidirectional manner [143].

After 72 h of co-cultivation, endothelial progenitor cells receive lysosomes from human umbilical vein endothelial cells, and vice versa, the lysosome transfer occurs under basal conditions [146]. When human umbilical vein endothelial cells are pretreated with glycated collagen type I, lysosomal dysfunction results, and the proportion of cells that engage in lysosomal exchange doubles [143]. Interestingly, the only factor contributing to the increase is lysosomal transfer from endothelial progenitor cells to human umbilical vein endothelial cells [146].

For instance, a malfunctioning lysosomal membrane cystine transporter called cystinosin causes cystinosis, a multisystemic lysosomal storage disease. Hematopoietic stem cell transplantation using lysosomal transplantation was described as a treatment for cystinosis [150]. After hematopoietic stem cells differentiate into macrophages, lysosomes containing the cystine transporters are transferred in both directions via tunneling nanotubes between fibroblasts deficient in cystinosis and macrophages derived from hematopoietic stem cells [150]. Engrafted hemopoietic stem cells and diseased proximal tubular kidney cells from mice with cystinosis formed a nanotubular structure [150]. To develop new methods of monitoring and visualizing this process, more research is necessary.

Many studies have indicated that lysosomal transfer plays a part in the therapeutic effects of stem cell-based therapies; however, it is unclear how other organelle transport contributes [142]. Similarly, it is unknown if stem cells can regenerate their pool of organelles after donation [142]. Additionally, more in vivo models could provide insight into the organelles and transport systems that could be artificially altered to maximize stem cells’ therapeutic potential [142].

##### Engineered Lysosomes

Intracellular compartmentalization and organization are vital characteristics of living cells. This organization allows a better synchronization of biological processes and specifies the role of subcellular organelles [3,141]. By careful understanding of the subcellular organization phenomenon, artificial organelles could be manufactured and mimic the natural organelle functional as a therapy to reestablish the function of living cells [141]. In this regard, using scaffolding proteins or assembling catalytically active protein complexes, like non-ribosomal peptide synthetase, are methods for simulating subcellular organelles [141]. The creation of catalytically active nanoscale components, such as lysosomes or other organelles, that can be incorporated into living cells to replace, fix, or enhance their functions is known as synthetic organelle manufacturing [141].

Artificial organelles were created using nanomedicine as an advanced kind of enzyme replacement therapy to treat metabolic disorders [141]. As a selective therapeutic approach in cancer treatment, artificial organelles are used to activate prodrugs in specific cells [141]. Although engineered organelles have the potential to give living cells new functions, this problem is still difficult to solve and has received little attention [141].

Synthetic biologists use DNA origami scaffolds or proteins with multiple binding motifs to create synthetic lysosomes [141]. The engineered lysosomes were fabricated and have digestion functions that promise to restore dysfunctional lysosomes [151]. Moreover, the enzymes combined with sustained nanoreactors were documented as artificial lysosomes [151]. These nanoreactors display catalytic activities similar to natural lysosomes [152]. Moreover, “nanozymes”, which are nanomaterials with enzyme-like properties, offer creative concepts for the construction of artificial organelles [152]. Multiple enzymatic activities, increased stability, ease of synthesis, reduced cost, and superior recyclability could all be achieved by creating artificial lysosomes [152].

## 4. Lysosomal Drug Targeting

**Figure 5:**
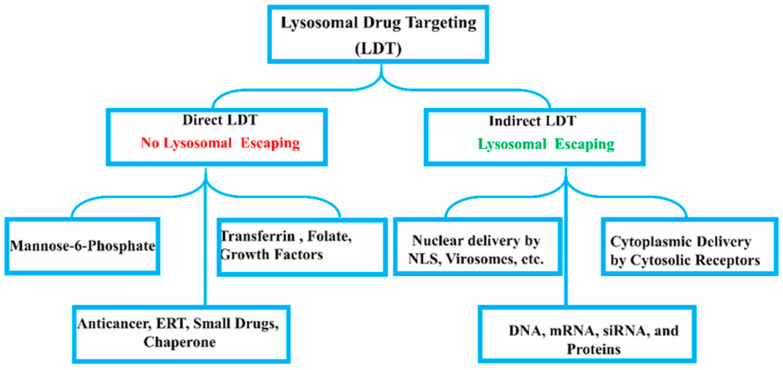
Approaches of lysosomal drug targeting.

Lysosomal drug targeting (LDT) is either a direct or indirect method. In the direct approach’s localization of drug cargoes into lysosomes, this is achieved by receptors-mediated endocytosis [3,30]. Several ligands such as M-6-P, TF, GF, and low-density lipoproteins were used for this purpose however they are imported into the lysosomes by clathrin-mediated endocytosis to achieve the lysosomal drug targeting [3,30]. On the contrary, indirect LDT could be achieved by nuclear or cytoplasmic drug delivery, afterward, the therapeutic effects are elicited at the lysosomal level. Indirect LDT requires endosomal escaping technology [3,30]. Figure 5 depicts the different approaches of LDT.

### 4.1. Direct Lysosomal Drug Targeting

The highly expressed clathrin-mediated endocytosis (CME) receptors on the cell surface specifically, M-6-P receptors, can be used for direct LDT [153]. Furthermore, ligands such as folate, transferrin, GF, and low-density lipoprotein are considered high-affinity ligands for CME [3,30]. These receptors are highly expressed on the cancer cell’s surface. Thus, the conjugation of drugs or carriers to these ligands could be utilized for specific tumor targeting [3,30]. Consequently, the conjugation of the drugs with these ligands, they were localized directly into the lysosomes [3,30]. Conjugated to transferrin, anticancer medications like doxorubicin and cisplatin exhibited greater cytotoxic activity than free drugs [153]. As well, the folate receptor is interesting in lysosomal cancer-targeting owing to its overexpression in many cancer cells surfaces. Folate and folate receptor substrates facilitate the binding of carriers conjugated to folate to the receptors due to a high affinity to folate receptors [153]. In this context, the intracellular uptake and cytotoxic activity of doxorubicin were enhanced by the liposomal formulation of doxorubicin modified with folate for folate receptor targeting in cells expressing folate receptors [154]. Likewise, Vitamin B12 and low-density lipoprotein are other ligands that can be used as drug conjugates for endosome targeting [154].

Interestingly, by functionalizing a delivery vehicle with targeting agents like proteins, peptides, lectins, antibodies, or aptamers unique to a marker in a diseased area, direct LDT can be achieved [153]. Direct LDT can be utilized for the treatment of LDs in a specific manner [4]. Moreover, nanotechnology is an effective approach for direct LDT [155,156]. After internalization of the nanocarrier imported by CME, they are trapped inside the endosomes. Then, the endosomes traffic toward and fuse with the lysosome to release the therapeutic agents [155,157,158].

### 4.2. Indirect Lysosomal Drug Targeting

#### 4.2.1. Cytosolic Delivery

The cytoplasm is the chief location for various biological processes such as metabolic pathways, protein biosynthesis, and other biological processes [3,7]. Moreover, the cytoskeleton, cytoplasmic receptors as well as subcellular organelles are located in the cell cytoplasm [3,7]. Thus, subcellular organelles such as the mitochondria, lysosomes, and nucleus were therapeutically affected by drug delivery to the cytosol [159]. In this context, vitamin D, or dexamethasone-labeled nanocarriers enhance the cytosolic delivery presence of their receptors present in the cytoplasm [159]. Moreover, nanocargoes could promote the delivery of small and macromolecule therapeutics such as DNA, mRNA, siRNA, and proteins to the cytoplasmic milieu [9,10,159]. Upon release of the cargoes, the content is in the cytosol neighbouring the translation machinery as well as the nucleus. The biological effect is done by modulating gene expression, gene knockout, or gene knockdown at proteomic, transcriptomic, or genomic levels [9,10]. Despite cytoplasmic drug targeting being an essential strategy for obtaining the maximum therapeutic index, however, it requires lysosomal escaping to avoid the degradation of therapeutic agents by lysosomal hydrolases and acidic milieu [4]. Most lysosomal therapeutics are small or macromolecules such as genes, gene editing tools, or proteins [4].

#### 4.2.2. Nuclear Delivery

The nucleus of the cell is the main site for the regulation of gene expression, and thus nuclear drug targeting is vital for organelles-associated disease correction and cell reprogramming [3,4,7]. Nuclear drug targeting by gene therapy or gene editing, radionuclides, chemotherapeutics, antibodies, and other therapeutic modalities could re-establish the cellular dysfunction [4]. The therapeutic agents could be delivered into the cell’s nucleus for correction of organelles malfunction such as lysosome or other organelles [3,7]. Small and macromolecule therapeutics such as DNA, mRNA, ncRNA, gene editing technology, and others can be delivered to the cell’s nucleus to modulate gene expression [10]. This way, nuclear drug delivery could be harnessed in cellular reprogramming, biotechnology, regenerative medicine, tissue engineering, and the treatment of lysosomal-associated diseases [10].

Specific basic peptide sequences called nuclear localization signals (NLS) exposed on the cargo bind to transport receptors [9]. NLSs are specific basic oligopeptides that have a high affinity for nuclear and importin-mediated nuclear transport [9]. Consequently, efficient nuclear transport of macromolecule therapeutics could be mediated by NLS for actual nuclear drug delivery [4]. Also, NLS-labeled nanoparticles loaded with therapeutic agents could be localized into the cell nucleus [4]. In this context, nanotechnology-based drug delivery strategies based on conjugation of NLS with nanocargoes could mediate peri-nuclear accumulation or direct insertion of therapeutics into the nucleoplasm [9].

It has been reported that genetic materials conjugated to NLS enhanced transfection efficiencies due to the electrostatic interactions of NLS with anionic DNA fragments [9]. Similarly, the cell-penetrating peptides have cationic natures, thus, they enhance intranuclear delivery due to NLS similarity [9]. Viral, none-viral, and extracellular vesicles are proposed to deliver codding nucleic acid and gene editing technology into the cell nucleus [9,10]. Taken together, nuclear drug targeting could be planned as an indirect approach for the restoration of organelles-associated malfunction including lysosome and other subcellular organelles.

The lysosomal degradation of the therapeutics is the main barrier to the intracellular import of drug cargoes into the intracellular milieu [3,8]. Thus, the escaping of lysosomal networks such as early endosomes, late endosomes, and proteasomes is essential for effective indirect lysosomal drug delivery [3,7]. However, most drugs or drug delivery systems are liable to degradation in the lysosomal network before reaching the cytoplasm or the nucleus [3,8].

Several approaches such as cationic penetrating peptides, pH-sensitive agents, proton sponge, and umbrella effect, besides using caveolin-mediated endocytosis could be harnessed to evade lysosomal network as a barrier for nuclear and cytoplasmic delivery [3,153]. In this context, a promising method for intracellular delivery of nucleic acids with endosomal escape capability is the use of cell-penetrating peptides [160,161]. Also, fusogenic peptides produced by influenza viruses improve siRNA’s endosomal escape to mediated gene silencing. Moreover, adenovirus-polylysine-DNA polyplexes improve intracellular DNA delivery by avoiding endosomes [153]. Amphipathic peptides facilitate the escape of DNA, siRNA, and proteins with lysosomal degradation [3]. These therapeutics are trafficking to the subcellular organelles to produce activity [153].

Additionally, polyethyleneimine and polyamidoamine dendrimers can prevent acidification of the lysosomes by pumping out protons [3]. This procedure causes endosomal disruption and makes it easier for medications to enter the cytosol [153]. Likewise, positively charged lipids cause the flip-flop process of the biomembranes to facilitate endosomal escaping [153]. Moreover, the escape of endocytosed molecules into the cytosol by conjugating the carrier with a photosensitizer stimulates the release of drugs after light activation [3]. For example, a photosensitizer used in cancer therapy is a dendrimer-based photosensitizer that localizes in the membrane of the endosomes [153].

### 4.3. Lysosomal Drug Delivery Systems

Ligands such as M-6-P, folate, transferrin, growth factors and others play an essential role in lysosomal drug delivery. In particular, M-6-P-tagged enzymes are created when lysosomal enzymes with a mannose moiety undergo phosphorylation in the cis-Golgi network [118]. M6P-R overexpression is thought to be an early indicator for a number of cancers, including melanoma, prostate, pancreatic, gastric, and hepatic breast cancers [118]. Consequently, lysosomes could be specifically targeted by the M-6-P receptor in drug delivery systems. In order to treat lysosomal-related disorders, M-6-P receptors may act as M-6-P conjugated carriers that facilitate the delivery of medication to lysosomes [118]. The selectivity and affinity of M-6-P ligands to M-6-P-R are an encouraged approach to develop an advanced lysosomal drug delivery system to improve lysosomal drug targeting [118]. Also, tri-N acetylgalactosamine is internalized intracellularly by M-6-P receptor and asialoglycoprotein receptor-mediated endocytosis to be localized in the lysosome [118]. The uptake of M-6-P-glycopolypeptide into the cytoplasm was demonstrated in the breast cancer cell line that overexpressed M-6-P receptors [162]. Moreover, create self-assembled nanocarriers with various shapes for lysosomal drug delivery by using amphiphilic M-6-P-glycopolypeptides [163,164]. Click reaction was used to create these FDA-approved enzyme-responsive or acid-responsive functionalizedM-6-P-glycopolypeptide and azide functionalized [163]. These enzyme- and pH-responsive cargoes could encapsulate hydrophobic drugs and internalize into the cells by endocytosis. The co-localization of these cargoes in the lysosomes was demonstrated by epifluorescence microscopy [163]. The clathrin-mediated pathway endocytosed the free polypeptides and their associated nanocarriers after they were absorbed by the cell [163]. Low-density lipoprotein receptors transport LDL-labeled cargoes to the lysosome [118]. Moreover, in order to break down extracellular cell membrane proteins linked to the lysosome-targeting receptors, lysosome-targeting chimera conjugates were developed [165]. Target moieties like antibodies and fragments that identify extracellular/cell membrane proteins with a ligand that lysosome targeting receptors recognize are present in these chimeras. M-6-P receptors and asialoglycoprotein receptors, which are involved in the transport of substances into particular cells’ lysosomes, have been utilized by the developed lysosome-targeting chimera technology [166].

The wide distribution of M-6-P receptors encourages drug delivery scientists to develop a tissue-specific lysosome-targeting chimera by harnessing other lysosome-targeting receptors [165]. Moreover, HepG2-specific asialoglycoprotein receptors are used to deliver antisense oligonucleotides and small interfering RNAs into the liver, which carry glycoproteins, including those of galactose or N-acetylgalactosamine, to the lysosome [166]. Furthermore, the developed lysosome-targeting chimeras would induce the degradation of several targets in lysosome-related diseases [166].

Nanomedicine developed nanoparticles, liposomes, protein aggresomes, and other lysosomal delivery cargoes for targeting the therapeutics in lysosomes [27]. In this context, liposomes have been shown to deliver medications for Batten disease and Fabry disease. EVs may be involved in inflammation, homeostasis, blood coagulation, angiogenesis, growth, cell differentiation, immunomodulation, apoptosis, stress response, and senescence because they transport bioactive molecules to their target cells. Numerous substances, including statins, calcium antagonists, and antioxidants, may influence the production of EVs [167]. In the last years, in order to deliver enzyme replacement therapy for Gaucher disease, EVs have been suggested as a bio-platform [27]. Likewise, EVs could deliver genetic materials (DNA or mRNA) and gene editing tools (miRNA or CRISPR-Cas9) into the cytosol or cell nucleus [9,10]. Besides, EVs conferring therapeutic protection, they can cross biological barriers such as blood-brain barriers and others [27]. Moreover, EVs are promising tools for lysosomal transplantation for the management of lysosomal defects. However, EVs containing membrane-bound organelles have been documented [31]. In this regard, because large EVs with organelles have the potential to be novel biomarkers for diagnosis and better therapeutic outcomes, they were expected to shed new light on clinical applications [168].

## 5. Conclusions and Future Outlook

The pathophysiological role of lysosomes in LSDs, LDs, aging, cancer, CVDs, NDDs, and various diseases was determined by this study. Lysosomes, on the other hand, are crucial regulators of human homeostasis that support immunological response, cell death, energy metabolism, and cell proliferation. Therefore, targeting lysosomes in certain human diseases is a practical, low-toxicity targeted strategy that can be further investigated for potential therapeutic intervention development. Treatment options for LDs may therefore include targeting the lysosomal membrane, acidification, cathepsins, calcium, mTOR, and autophagy. As well, restoration of lysosomal defects by small molecule therapy, enzymes, genes, and gene editing could cure LDs. Moreover, senotherapeutics, endosymbiotic therapy, lysosomal transplantation, and artificial lysosomes are promising approaches for controlling the dilemma of LDs. The LDT maximizes the therapeutic efficacy and safety of small and macromolecule therapeutics for LDs. Thus, Optimizing LDT can enhance the therapeutic outcomes for LDs, cancer and age-related diseases. M6P ligand targets lysosomes by binding to the M6P receptor. Nanocarriers and extracellular vesicles are promising tools for LDT lysosomal transplantation for the managing of LDs. Several small, macromolecule therapeutics and senotherapeutics are approved and delivered to lysosomes for LD management. However, endosymbiotic therapy, lysosomal transplantation, and artificial lysosomes are still in the experimental stages and they have ethical and biosafety issues.

## Figures and Tables

**Table 2:** Examples of lysosomal-acting medicines with their mechanism of action and medical applications.

Drug	Mechanism of Action	Applications
Chloroquine	Affect lysosomal pH Alters the lysosomal permeability Inhibitor of autophagic signals	Antiparasitic Anti-inflammatory Antiviral
Hydroxychloroquine	Affect lysosomal pH Alters the lysosomal permeability Inhibitor of autophagic signals	Antiviral effect Anti-inflammatory Autoimmune disorders
Suramin	Inhibiting ATP signaling Inhibitor of lysosomal enzymes Suppressing phagosome-lysosome fusion	Antiparasitic drug for treatment of trypanosomiasis
Metformin	AMPK activation Inhibition of mTOR Inhibitor of autophagic flux Block the autophagosome formation	Aging Diabetes Cancer Senolytic agent
Trehalose	Inducer of TFEB mTOR Inducer of autophagy	Neurodegeneration diseases
Imipramine	Inhibition of sphingomyelinase Implicated in autophagy in neurons	Antidepressant
Amiodarone	Inhibits autophagic Increased autophagic flux Induced apoptosis	Antiarrhythmic
Azithromycin	Excessive lysosomal ion-trapping. Blockade of autophagy	Infections
Trichostatin A	mTOR blockage	Cardiopathy
Salermide	Inhibitors of mTOR pathway	Lung cancer
Valproic acid	Inhibitors of HDAC1/AKT signaling	Gastric cancer
Ibrutinib	Phosphorylation	Cancer
Fasudil	Affects AKT-mTOR pathway	Traumatic urethral Attack

**Table 3:** Examples of some small-molecule and macromolecule FDA-approved therapeutics for the treatment of lysosomal diseases.

Medicine	Class/Mechanism	Specific Application in LSD
Migalastat hydrochloride	Small-molecule therapy, SRT	Fabry disease
Cysteamine	Small-molecule therapy, SRT	Cystinosis
Eliglustat	Small-molecule therapy, SRT, ERT	Gaucher disease type I
Miglustat	Small-molecule therapy, SRT, ERT	Gaucher disease type III
Venglustat	Small-molecule therapy, SRT	Fabry disease
Aglusidase Alpha	Macro-therapy, ERT	Pompe disease
Cerliponase Alpha	Macro-therapy, ERT	The neuronal ceroid lipofuscinoses
Elosulfase Alpha	Macro-therapy, ERT	MPS IV A
Vestronidase Alpha	Macro-therapy, ERT	MPS VII
Agalsidase Beta	Macro-therapy, ERT	Fabry disease
Sebelipase Alpha	Macro-therapy, ERT	Acid lipase deficiency
Arylsulfatase B	Macro-therapy, ERT	MPS VI
Iduronate sulfatase	Macro-therapy, ERT	MPS II
Alpha-L-iduronidase	Macro-therapy, ERT	MPS I
Glucocerebrosidase	Macro-therapy, ERT	Gaucher disease Type 1
Alpha-galactosidase A	Macro-therapy, ERT	Fabry Disease

## Data Availability

All data are embedded in the manuscript.
